# Modulatory effects of cancer stem cell-derived extracellular vesicles on the tumor immune microenvironment

**DOI:** 10.3389/fimmu.2024.1362120

**Published:** 2024-06-19

**Authors:** Xinyu Li, Cuilian Zhang, Wei Yue, Yuening Jiang

**Affiliations:** ^1^ Department of Animal Science, College of Animal Science, Hebei North University, Zhangjiakou, Hebei, China; ^2^ Department of Gynecology and Obstetrics, Key Laboratory for Major Obstetric Diseases of Guangdong Province, Key Laboratory of Reproduction and Genetics of Guangdong Higher Education Institutes, The Third Affiliated Hospital of Guangzhou Medical University, Guangzhou, Guangdong, China; ^3^ Reproductive Medicine Center, Henan Provincial People’s Hospital, Zhengzhou University, Zhengzhou, China; ^4^ State Key Laboratory of Female Fertility Promotion, Center for Reproductive Medicine, Department of Obstetrics and Gynecology, Peking University Third Hospital, Beijing, China; ^5^ National Clinical Research Center for Obstetrics and Gynecology, Peking University Third Hospital, Beijing, China; ^6^ Key Laboratory of Assisted Reproduction, Peking University, Ministry of Education, Beijing, China

**Keywords:** cancer stem cells, extracellular vesicles, exosomes, immune cells, tumor microenvironment

## Abstract

Cancer stem cells (CSCs), accounting for only a minor cell proportion (< 1%) within tumors, have profound implications in tumor initiation, metastasis, recurrence, and treatment resistance due to their inherent ability of self-renewal, multi-lineage differentiation, and tumor-initiating potential. In recent years, accumulating studies indicate that CSCs and tumor immune microenvironment act reciprocally in driving tumor progression and diminishing the efficacy of cancer therapies. Extracellular vesicles (EVs), pivotal mediators of intercellular communications, build indispensable biological connections between CSCs and immune cells. By transferring bioactive molecules, including proteins, nucleic acids, and lipids, EVs can exert mutual influence on both CSCs and immune cells. This interaction plays a significant role in reshaping the tumor immune microenvironment, creating conditions favorable for the sustenance and propagation of CSCs. Deciphering the intricate interplay between CSCs and immune cells would provide valuable insights into the mechanisms of CSCs being more susceptible to immune escape. This review will highlight the EV-mediated communications between CSCs and each immune cell lineage in the tumor microenvironment and explore potential therapeutic opportunities.

## Introduction

Cancer stem cells (CSCs), also known as tumor initiating (propagating) cells, despite constituting a relatively small population of the tumor cells, play a pivotal role in fueling tumor growth due to their self-renewal and multi-lineage differentiation ability (1–4). This concept, introduced several decades ago, has stimulated extensive studies aimed at decoding the clinical observations through CSCs (5–9). Patients who show initial partial or complete remission through anti-cancer treatments by chemotherapy or radiation may eventually experience tumor relapse or metastasis, conditions that tend to be markedly intractable owing to the increased resistance to therapies (10, 11). This phenomenon is likely attributed to CSCs’ resilience against the therapeutic regimens. Besides, CSCs also contribute to intratumoral heterogeneity (ITH) by differentiating to all kinds of cancer cells with different phenotypic features, some of which can disseminate to other parts of the body (12–14).

CSCs intricately interact with cancer supporting cells especially immune cells in the tumor ecosystem, sculpting a conducive niche and employing mechanisms for immune evasion and immunosuppression, including activating immune escape pathways and suppressing antigen processing and presentation proteins (15). CSCs also overexpress programmed cell death 1 ligand 1 (PD-L1) on the cell surface, an incredibly important immune checkpoint protein that counteract the antitumor immune response (16, 17). Unraveling how CSCs engage with cancer-associated immune cells is gaining increasing attention, as these interactions hold considerable potential as immunotherapeutic targets.

Extracellular vesicles (EVs) are small lipid bilayered membrane vesicles composing of two main subgroups, exosomes and microvesicles (MVs) (18–20). These vesicles range in size from a few tens of nanometers to multiple micrometers (21, 22). Secreted by all cell types, EVs are responsible for the transfer of functional biological cargos including nucleic acids, proteins, and lipids between cells, mediating critical intercellular communication (23, 24). EVs are irreplaceably involved in modulating various innate and adaptive immune processes including antigen presentation, activation of T cells and B cells (25–27). Within the tumor ecosystem, EV-mediated communication is preferentially characterized by EVs produced by CSCs being internalized by other cells, a process integral for the dissemination of CSC-specific traits, which is essential for shaping the tumor immune microenvironments. For example, pancreatic CSC-derived EVs carry agrin protein to increase YAP activation to promote tumor cell proliferation (28). EVs from colorectal CSC could transfer metastatic properties to the non-CSCs via miR-200c (29). CSC-derived EVs are likely to interact with immune cells enriched in the CSC niche, including MHC-II expressing macrophages and programmed cell death 1 (PD1) positive T cells, potentially promoting tumor progression through immunosuppression (30). Conversely, immune cell-derived EVs enhance CSC stemness and propagation (31–33). The reciprocal transfer of EVs between CSCs and immune cells operate in concert to create a tumor-supporting niche.

In this review, we will summarize and discuss the recent findings on the biological functions of CSC-derived EVs, with a focus on their immunological role through engagement with various types of tumor-associated immune cells, and how EV-mediated interactions between CSCs and immune cells contribute to shaping the tumor immune microenvironments.

## Overview of CSCs

ITH is a major obstacle in cancer treatment, leading to aggressive tumor growth and treatment failures (34–36). Elucidating the sources of ITH is of great significance to overcome therapy resistance. ITH present in two forms, spatial heterogeneity, which involves the unequal distribution of tumor subpopulations across different regions, and temporal heterogeneity, which refers to genetic diversity within a tumor over time (34, 37). Technological advances such as multiregional sampling, liquid biopsy, single-cell sequencing, and spatial transcriptome provide insights into decoding the intricate compositions of ITH (38–40). Single-cell transcriptomic analysis of various types of tumors have uncovered distinct subpopulations of tumor cells, each characterized by varying levels of differentiation and stemness (41–44). Of note, various driving forces are emerging to be responsible for ITH, encompassing genome instability and clonal evolution (45, 46), metabolic adaptation (47), epithelial-mesenchymal transition (EMT) (48) as well as environmental factors such as hypoxia (49) and inflammation (50). Among these, the existence of CSCs plays a pivotal role in the development and maintenance of ITH (13, 51).

**Figure 1:**
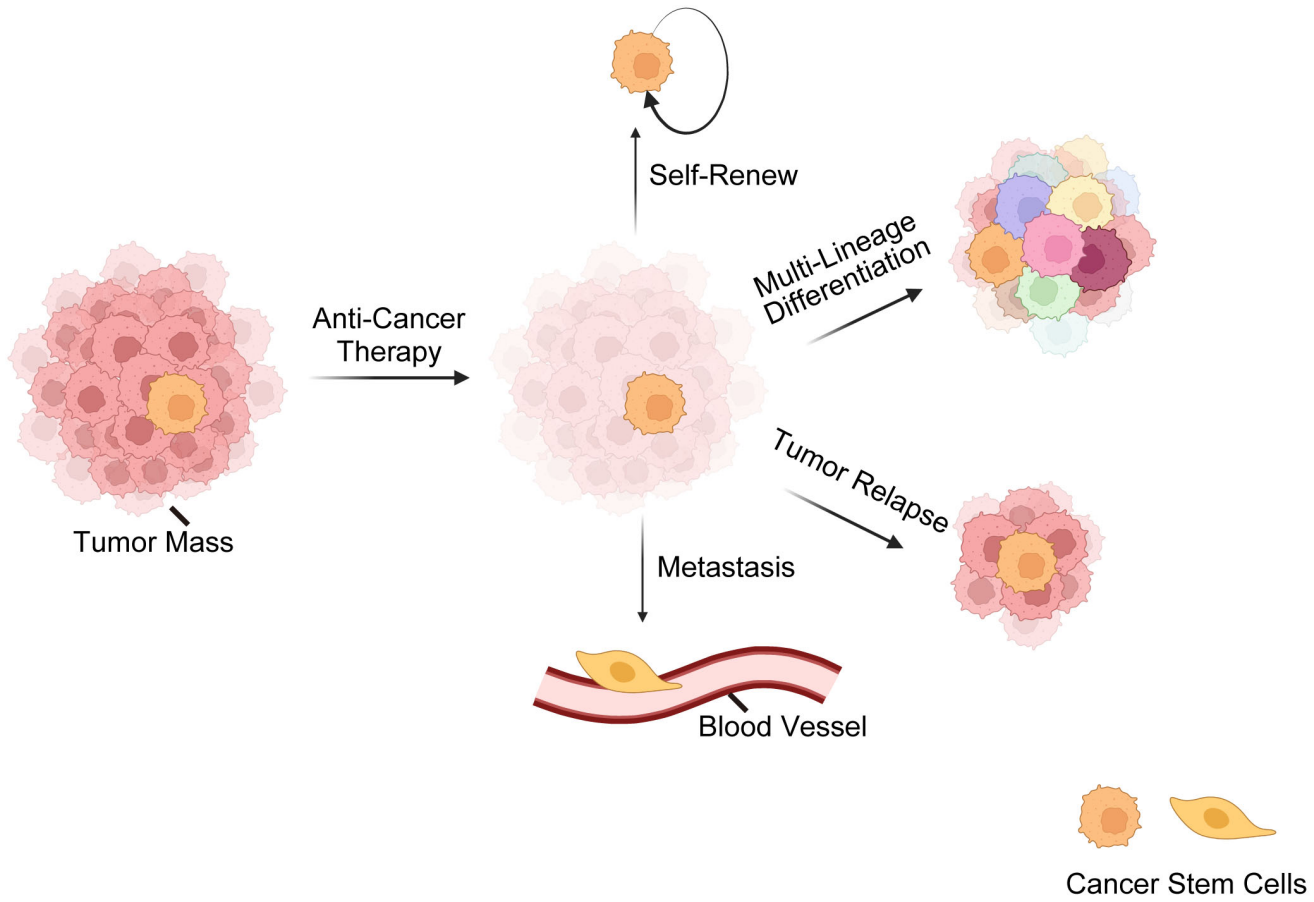
Overview of cancer stem cells (CSCs). This illustration delineates the fundamental characteristics of CSCs, encompassing their resilience against standard anti-cancer treatments. CSCs possess a remarkable ability for self-renewal, ensuring the perpetuation of the cancer cell population. CSCs are also capable of multi-lineage differentiation, which contributes to the cellular complexity and heterogeneity of tumors. Furthermore, CSCs are often implicated in tumor relapse due to their ability to remain dormant and then re-initiate tumor growth. Lastly, their role in metastasis is underscored by their potential to disseminate and form new tumors at distant sites, which is a hallmark of advanced cancer stages. These CSC characteristics are critical for understanding tumor behavior and developing targeted therapeutic strategies.

Here comes the concept of CSCs, a subset of tumor cells capable of self-renewal, tumor-initiating, multi-lineage differentiation, therapy-resistance, metastasis, relapse, etc. (7, 52–54) (**Figure 1**). The CSC theory posits that tumors are hierarchically structured, with CSCs at the top (7, 55–57). Nevertheless, recent findings (2, 13, 58) have revealed that CSCs and non-CSCs are dynamic and can transition between states in response to specific stimuli, complicating tumor eradication. Initially identified in hematological malignancies, CSCs are now recognized in various solid cancers (59–62). Three hypotheses explain CSC origins (63, 64): transformation of non-cancerous stem cells through a series of oncogenic mutations (65), acquisition of pluripotency by progenitor cells (66), and dedifferentiation of differentiated cells (67).

Certain surface markers such as CD133, CD44, epithelial cell adhesion molecule (EPCAM), and intracellular markers such as aldehyde dehydrogenase (ALDH) have been identified for CSCs (68–72). ALDH is an enzyme mediating aldehyde detoxification, which is assessed by the ALDEFLUOR assay, is instrumental in drug resistance (73–75). However, these markers are not solely specific to CSCs, and some CSCs don’t exhibit these markers at all (9, 76, 77). Moreover, CSCs typically constitute less than 1% of the tumor mass, and such scarcity further complicates their isolation and identification (77).

Besides markers, the characterization of CSCs requires surrogate functional assays (78–80). Current well-known surrogate methods include *in vitro* tumorsphere formation and *in vivo* limiting-dilution tumorigenicity assays (81–83). *In vitro* tumorsphere formation assay evaluates the ability of cells to grow and form spheres in a three-dimensional, anchorage-independent culture environment (84, 85). *In vivo* limiting-dilution tumorigenicity assay, which tests the tumor-initiating capacity of cells by transplanting them into immunocompromised mice and observing tumor formation, is considered the gold standard for CSC research (86, 87).

CSCs contribute to therapy resistance through several mechanisms (88–90), including high levels of multi-drug efflux ATP-binding cassette transporters, slow-cycling state, enhanced DNA repair capacity, apoptosis evasion, immune-privileged property, etc. Furthermore, EMT activation is tightly associated with the formation of CSCs (91). Consequently, CSCs consist of heterogenous subtypes occupying different locations and exhibiting varied EMT characteristics within the primary tumor (92, 93), further complicating their therapy resistance.

Additionally, it is important to emphasize that CSCs have been found to actively reshape the tumor microenvironment into immunosuppressive state, facilitating their own growth and proliferation while evading the therapeutic elimination (15, 94, 95). This interaction is significantly mediated by EVs, powerful cell-cell communicators that plays a crucial role in modulating the immune microenvironment (96). Through EVs, CSCs transferring a diverse array of biological cargos to surrounding or distant immune cells, eliciting immunosuppressive responses that protects CSCs and fosters tumor progression (28).

## The landscape of EVs: classifications and molecular constituents

**Figure 2:**
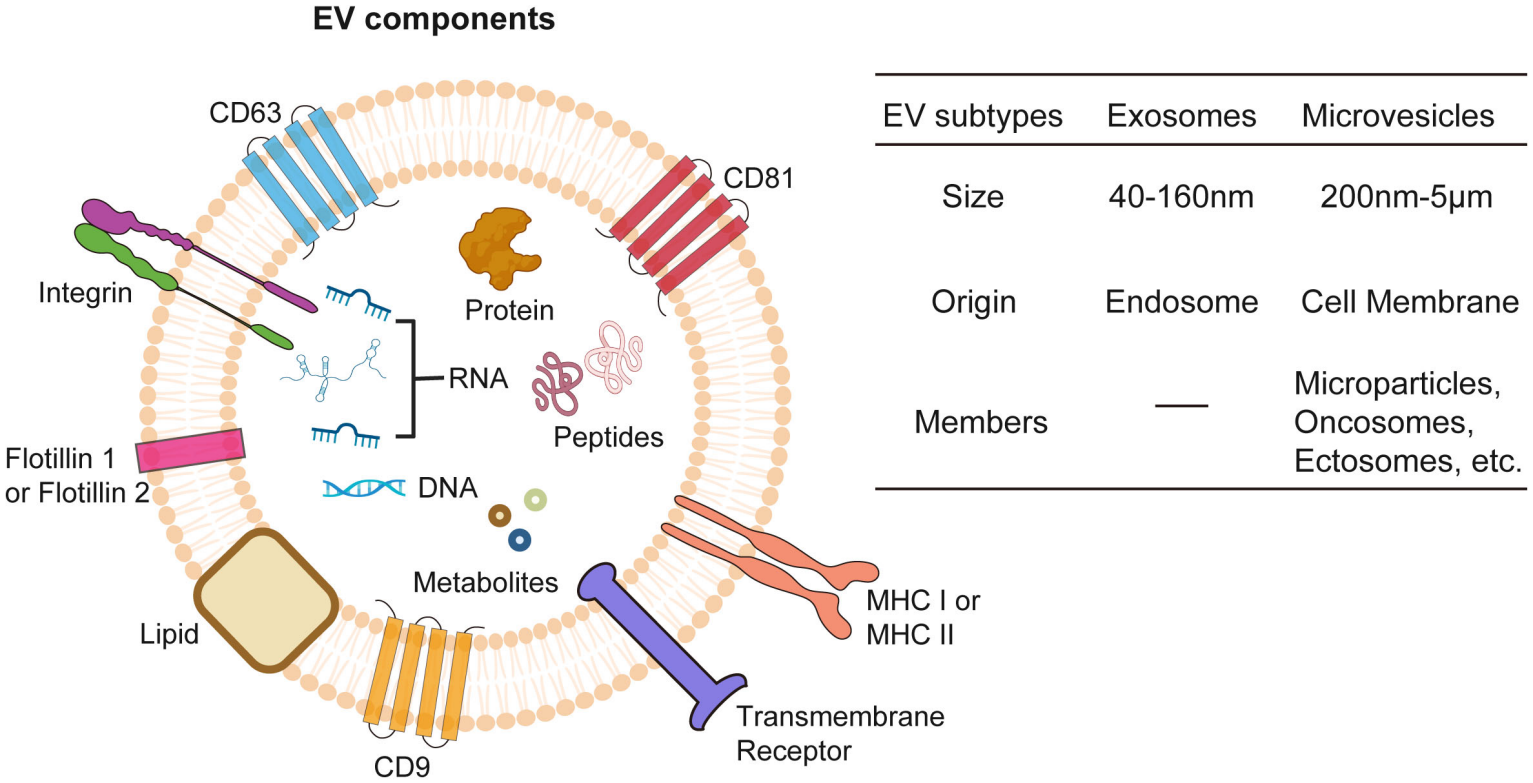
Extracellular vesicles (EVs). EVs encapsulate a diverse array of bioactive molecules, including surface proteins, cytosolic proteins, nucleic acids (both DNA and RNA), metabolites, lipids, and peptides. EVs are classified into exosomes and microvesicles based on origin of formation and their size.

EVs are lipid-bilayer membrane structures secreted by virtually all living cells, encompassing two main subtypes, exosomes and MVs, whose sizes ranging from about 50 nm to 5 µm (97, 98) (**Figure 2**). EVs contain cellular bioactive components like proteins, lipids, metabolites, and nucleic acids, reflecting their cell of origin and functioning as mediators of intercellular communication (99, 100). EVs perform multifaceted functions including waste disposal, signal cargo delivery to alter recipient cell physiology, and mediating interactions between cells and extracellular matrix (101–105). Furthermore, they can also facilitate long-distance communication via blood or lymph (106, 107). Separating different EV subtypes is challenging due to overlapping properties, with methods like differential centrifugation, size-exclusion chromatography, and immunoprecipitation used in combination to improve specificity (108).

Exosomes stand out as the most well-studied subtype of EVs with a size ranging between 40–160nm, primarily due to their unique biogenesis, small size, and specific molecular content (109). Exosomes play a pivotal role in cellular communication and cancer research. Originating from endosomal compartments within cells, they carry an array of biomolecules which reflect their cellular origin and can influence recipient cell behavior. This makes them key players in tumor progression and ideal for targeted drug delivery and biomarker discovery in cancer diagnostics and therapeutics (110–112). Their small size, specific content, and stability in bodily fluids enhance their potential, making them promising candidates in the medical and scientific exploration of cancer.

Contrasting with exosomes, which originate from endosomal pathways, MVs are formed by budding directly off the plasma membrane and are generally larger, with sizes spanning from 200–5000 nm (97, 113). MVs refer to a diverse group of membrane-derived vesicles, including microparticles, oncosomes, or ectosomes, and is less characterized EV subtype whose cargo trafficking mechanisms are still under investigation (114). Besides functional cargos as RNAs, proteins, lipids, and metabolites, MVs could also mediate the transfer of mitochondria between cells (115), which boosts ATP production in the recipient cells. Studies have shown that cancer cell-derived MVs participate in tumor progression (116, 117), while MVs from radiation treated cancer cells exert antitumor effect through immunogenic death pathway (118). These findings suggest a promising functional role of MVs in cancer therapeutics.

### CSC-derived EVs in oncogenic events

EVs generated from CSCs are instrumental in enhancing tumorigenesis, advancing metastasis, and fostering therapy resistance across various types of cancers by transferring associated vicious traits indicative of donor cell (29, 119–122) (**Table 1**). Colorectal CSCs secrete EVs enriched with glycoprotein CD147 can subsequently trigger signaling pathways associated with tumorigenesis in recipient cancer cells (128). Besides, miR-200c in EVs from colorectal CSCs could convey metastatic traits to accelerate tumor progression (29). In triple-negative breast cancer, CSCs release EVs that can stimulate specific cancer-associated fibroblasts and remodel endothelial cells, accelerating invasiveness and preparing distant metastatic niches (129). Lung CSCs could transfer their strong metastatic properties to the whole tumor mass through exosomal lncRNA Mir100hg/miR-15a-5p/glycolysis pathway (130). Gastric CSCs induce tumor cells to gain malignant and metastatic behaviors and stemness features via EVs internalization (131), possibly due to a gastric CSCs marker gene DCLK1, which could transfer the migratory property to the recipients (132). Melanoma CSCs secrete EVs that enhance metastatic ability of non-stem cancer cells via miRNA-592, which activates the MAPK/ERK signaling pathway (125). Breast CSC-derived EVs carry ARRDC1-AS1 to promote breast cancer malignancy by modulating miR-4731–5p/AKT1 axis to foster tumor growth and aggressiveness (123). In addition, CSCs secrete certain tumor suppressors out the cells by EVs. Acute myeloid leukemia stem cells secrete more miR-34c-5p out to attenuate senescence through RAB27B-mediated exosome trafficking (133).

**Table 1:** CSC-derived EVs in oncogenic events.

Cancer type	EV cargo	Function	Reference
Breast cancer	ARRDC1-AS1	Promote the malignant progressive phenotypes via the miR-4731–5p/AKT1 axis.	(123)
	miR-197	Promote epithelial-mesenchymal transition thus increase BC cells proliferation and metastasis	(124)
HNSCC	_	EVs have a selective impact on specific immune cells to modulate anti-cancer immune response	(30)
Pancreatic cancer	Agrin protein	Promote YAP activity via LRP-4 to contribute to tumor progression	(28)
Melanoma	miR-592	Increase the metastatic ability of MPCs via miR-592/PTPN7/MAPK axis	(125)
Colorectal cancer	miR-200c	Enhance invasion, metastasis and stemness associated with PI3K/Akt/mTOR activation	(29)
Ovarian cancer	_	Promote the migration ability and pro-tumorigenic phenotype MSCs	(126)
NSCLC	APE1 shRNA	Reverse Erlotinib resistance of NSCLC via inhibiting IL-6/STAT3 signaling	(127)

HNSCC, head and neck squamous cell carcinoma. NSCLC, non-small cell lung cancer.Symbol “-” indicates that the corresponding study did not specify the function at the EV cargo level, but rather at the whole EV level.

EMT and angiogenesis are essential mechanisms of tumor metastasis (134, 135), with CSC-derived EVs engage in the regulation of these processes. Renal CSC-derived exosomes, carrying miR-19b-3p, trigger EMT in renal tumors and enhance distant metastasis (136). EVs from glioma CSCs containing vascular endothelial growth factor A (VEGF-A) significantly boost angiogenesis and increase vascular permeability in brain endothelial cells, indicating significant contribution of CSC-derived EVs to the tumor’s vascular development (137, 138).

### CSC secretome and immune modulation

The concept of secretome, now updated to extend beyond merely proteins, have led to the recognition of EVs as the nanostructured/microstructured secretome, composing a complex assembly of bioactive molecules with significant implications for intracellular communications and dynamics inside the tumor microenvironment (TME) (139–141). Furthermore, CSC secretome covers a diverse spectrum of bioactive molecules released out of cells, including various soluble factors like growth factors, cytokines, chemokines, and proteins (142–144). Delineating the roles these secretome compositions play on immune interactions will provide an integrated understanding of crosstalk between CSC and immune cells, setting the stage for the subsequent sections that focus on the specific roles of EVs in this interplay.

CSC secretome has profound impact on tumor growth and TME modulation (**Table 2**). Secretome profiles of melanoma CSCs include proteins enriched with cell proliferation, cell survival and negative regulation of apoptosis (145). Breast CSCs actively secrete CXCL1, a chemokine that plays a crucial role in stimulating their proliferation and enhancing their capacity for self-renewal, contributing significantly to the progression and aggressiveness of breast cancer (146). Glioma CSCs generate and release immune cytokines such as soluble colony-stimulating factor (sCSF-1), transforming growth factor (TGF)-β1, C-C motif chemokine 2 (CCL2), VEGF, macrophage inhibitory cytokine-1 (MIC-1), and galectin-3 into TME, contributing to the suppression of innate immunity characterized by the induction of immunosuppressive macrophages and regulatory T cells and effector T cell apoptosis (147, 148). CSCs release macrophage migration inhibitory factor (MIF), which binds with C-X-C motif chemokine receptor 2 (CXCR2) presenting on myeloid-derived suppressor cell (MDSCs), leading to production of arginase 1, thereby suppressing CD8+ T cells (150). Also, previous studies revealed that the glioma CSCs could trigger B7-H4 expression in both tumor and immune cells through IL6-STAT3 pathway activation and stimulate PD-L1 expression within the TME (147, 149). These cytokines, chemokines and immune checkpoint proteins work synergistically to lead to immunosuppression.

**Table 2:** Biological functions of CSC secretome.

Donor cell	Secreted molecules	Function	Reference
Melanoma CSCs	Proteins related to cell proliferation and survival	Sustain cell survival, while Theo supplement induce CSC differentiation	(145)
Breast CSCs	CXCL1	Stimulate CSC proliferation and enhance self-renewal	(146)
Glioma CSCs	sCSF-1, TGF-β1, CCL2, VEGF, MIC-1, galectin-3	Induce phenotypes of Treg and immunosuppressive macrophage, and stimulate effector T cell apoptosis	(147–149)
	MIF	Suppress CD8+ T cell activity	(150)

## Exploring EV-mediated interactions between CSCs and immune cells in the TME: implications for tumor immunity

**Figure 3:**
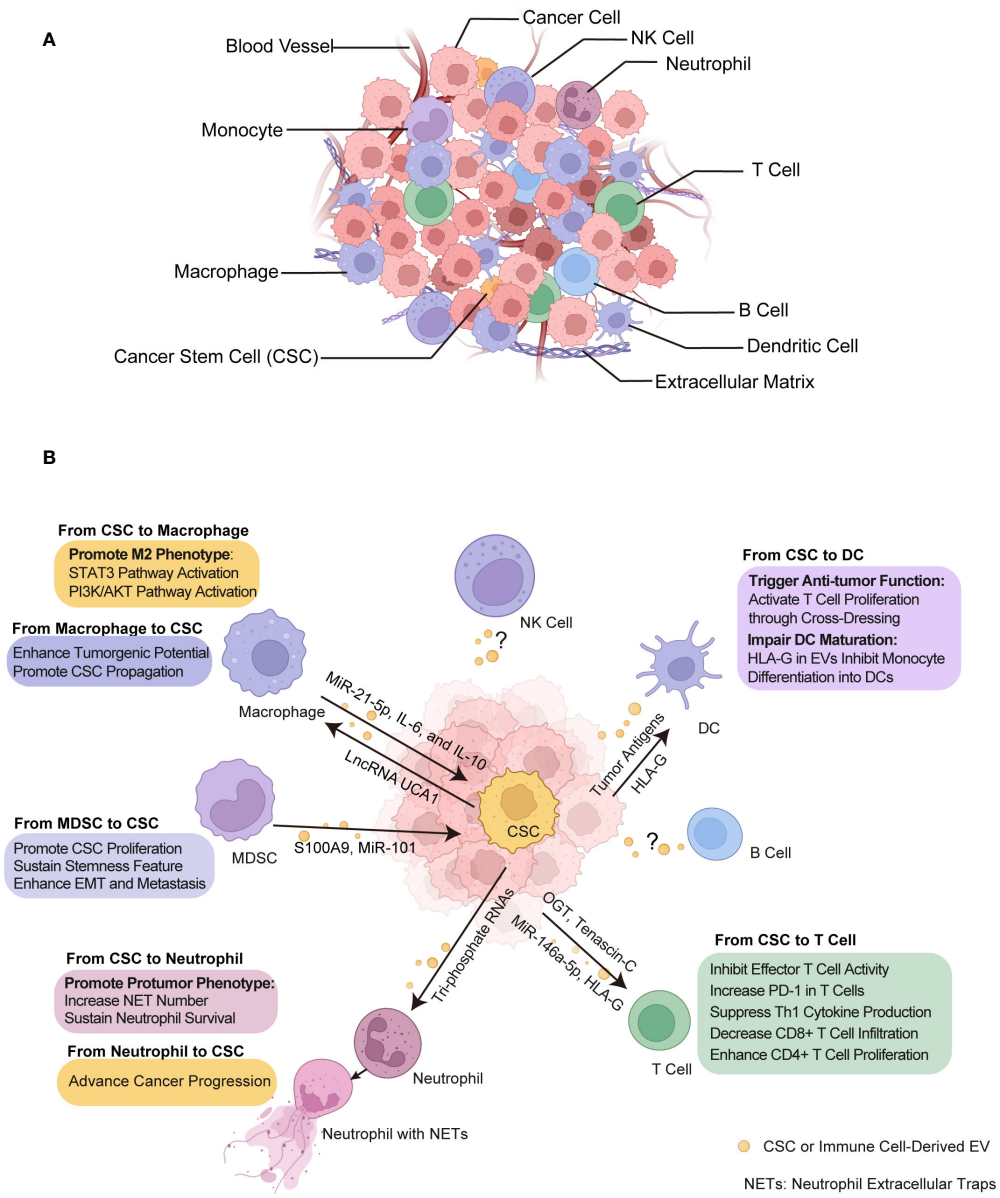
Crosstalk between CSCs and immune cells through EVs within tumor microenvironment (TME). **(A)** TME composition. The tumor ecosystem encompasses a diversity of cell types including cancer cells, CSCs, immune cells, and non-cellular component like extracellular matrix and blood vessels, all of which are integral to TME. **(B)** The effect of EVs on CSCs and immune cells. These EVs transport biological signals that prime immune cells to undergo various functional alterations such as immunosuppressive phenotype acquisition, cytotoxicity inhibition and DC activation, and in turn, immune cells exert certain influence on CSCs such as promotion of growth and metastasis. CSC, cancer stem cell. NETs, neutrophil extracellular traps. DC, dendritic cell. MDSC, myeloid-derived suppressor cell. Some elements in **Figures 1**–**3** were created with BioRender.com.

The TME is a complex and dynamic battlefield consisting of cancer cells, CSCs and cancer supporting cells, serving as a critical zone for the interplay between immune cells and CSCs (**Figure 3A**). This environment is a hub where diverse immune cells from both innate and adaptive immune systems converge (151, 152). Dendritic cells (DCs) are key in antigen presentation and the initiation of immune responses, while macrophages, also involved in antigen presentation, display ambiguous effects on tumors by either fostering or inhibiting their growth. MDSCs predominantly suppress immune activity, facilitating tumor immune evasion. Natural killer (NK) cells, adept at autonomously destroying cancer cells, and neutrophils, whose impact on cancer can vary from hindering to promoting tumor progression. In terms of the T cell population, subsets including cytotoxic T lymphocytes (CTLs) are directly responsible for recognizing and eradicating cancer cells, highlighting their critical role in antitumor immunity. T helper 17 (Th17) cells, a subset of CD4+ helper T cells, known for their secretion of interleukin-17 (IL-17), exhibit a dual role in cancer by either promoting inflammation that can support tumor growth or recruiting effector T cells, NK cells and DCs into TME that enhance antitumor responses.

Mounting evidence have shown that EVs released by cancer cells, especially CSCs, are capable in modulating both innate and adaptive immune responses, thereby facilitating to establish pro-tumorigenic and pro-metastatic immune niches through their interactions with various immune cell types within TME (153–156). Understanding the diverse functions and interactions of these immune cells with CSCs through EVs provides insight into the complex nature of cancer and opens avenues for innovative therapeutic strategies (**Figure 3B**, **Table 3**).

**Table 3:** EV communications between CSCs and immune cells.

Donor cell	Recipient cell	EV cargo	Function	Reference
Glioblastoma CSCs	Macrophages	STAT3 pathway components	Induce M2 macrophage phenotype	(157)
Oral CSCs	Macrophages	lncRNA UCA1	Induce M2 macrophage phenotype	(158)
HNSCC	M2 macrophage and PD1+ T cells	—	Promote immune evasion	(30)
Macrophages	Pancreatic CSCs	miR-21–5p	Promote CSC stemness	(33)
Macrophages	Ovarian CSCs	IL-6, IL-10	Promote CSC proliferation	(31)
Colon CSCs	DCs		Activate T cell proliferation	(159)
Renal CSCs	DCs and T cells	HLA-G	Inhibit DC maturation and T cell function	(160)
Colon CSCs	Neutrophils	Tri-phosphate RNAs	Sustain neutrophil survival and recruit them to advance cancer progression	(161)
Melanoma CSCs	Neutrophils	—	Increase pro-tumor effect of neutrophils	(162)
MDSCs	Ovarian, breast and pancreatic CSCs	—	Promote CSC stemness and propagation	(163–165)
MDSCs	Colorectal CSCs	S100A9	Promote CSC stemness and survival	(32)
Esophageal CSCs	T cells	OGT	Increase PD-1 in T cells to be immunosuppressive	(166)
HNSCC CSCs	PD1+ T cells	—	Promote immune escape	(30)
Brain CSCs	T cells	tenascin-C	Inhibit T cell-induced immune response	(167)
Colorectal CSCs	T cells	miRNA-146a-5p	Decrease CD8+ T cell infiltration	(168)
Glioma CSCs	T cells	—	Inhibit T cell proliferation, activation and Th1 cytokine production	(169)

Symbol “-” indicates that the corresponding study did not specify the function at the EV cargo level, but rather at the whole EV level.

### Macrophage

Tumor-associated macrophages are a diverse group of macrophages usually originating from circulating monocytes, recruited to TME (170). In some solid tumors, macrophages can constitute more than 50% of the tumor mass (171, 172), and the abundance of infiltrated macrophages usually associated with distinct clinical prognosis (173). Contrary to the notion of them being a homogenous population, macrophages exhibit a wide range of behaviors and characteristics, influenced by the specific type, stage, and immune context of the tumors they infiltrate (174). This variability extends to their roles, which are generally categorized into two subpopulations: classically activated (M1 or M1-like) and alternatively activated (M2 or M2-like) macrophages (175). Macrophages engage in mutual interactions with tumor cells and other cells like platelets, neutrophils, and various T cells, while also suppressing NK and CD8+ T cell activation. These interactions present numerous targets for therapies aimed at promoting an anti-tumor response (176). Emerging research has unveiled intricate communication pathways involving CSC-derived EVs that orchestrate a complex interplay with macrophages in various cancer types, profoundly supporting the immunosuppressive microenvironment and tumor progression (30, 157, 158).

CSC-derived EVs promote macrophage to exhibit M2 phenotype. Glioblastoma CSC-generated exosomes (GDEs) preferentially target monocytes to promote their conversion into immunosuppressive M2 macrophages within TME, a process characterized by upregulated PD-L1 expression due to the components of the STAT3 pathway carried by these GDEs (157). Oral squamous CSC-derived small EVs transport the lncRNA UCA1 which, by sequestering miR-134, modulates the PI3K/AKT pathway via LAMC2 to drive macrophages toward an immunosuppressive M2 phenotype, thus promoting tumor growth and inhibiting T-cell function (158). CSC-derived EVs in head and neck squamous cell carcinoma (HNSCC) specifically interact with M2 macrophages and PD1+ T cells, crucial immune constituents enriched in CSC niche, contributing to immunosuppression landscape that impedes effective HNSCC therapy (30).

The EV communication routes can be bidirectional and reciprocal between CSCs and macrophages, with M2 macrophage-derived EVs enhancing the tumorigenic potential of pancreatic CSCs. This enhancement is mediated through the transfer of miR-21–5p, which suppresses KLF3 expression to promote stemness (33). Furthermore, activated M2 macrophages promote ovarian CSC propagation through IL-6 and IL-10 cytokine secretion in TME (31).

### DC

DCs, as professional antigen-presenting cells, play a pivotal role in the immune response within the TME. They are essential in capturing foreign antigens and presenting them to T cells via multiple ways including direct, cross-presentation and cross-dressing (177), thereby activating the adaptive immune system to mount an effective killing against tumors. Specifically, EVs also have the capacity to deliver tumor antigens to DCs, a phenomenon known as cross-dressing, which has garnered significant recent attention in research (178). DCs can activate T cell proliferation by co-culture with colon CSC-derived exosomes, possibly due to the increased ratio of IL-12 to IL-10 (159).

However, the cargo of CSC-derived EVs not only transfer tumor antigens for immune activation but deliver a diversity array of functional cargo that actively hinder DC function as well. A study focusing on renal CSC-derived EVs, particularly those expressing CD105, significantly disrupt the maturation of monocyte-derived DCs and the activation of T cells. Notably, this disruption is more pronounced than the situations in non-stem tumor cells. This immune escape effect is largely attributed to the expression of human leukocyte antigen (HLA)-G by the CSCs, which is then packaged and released by EVs (160).

### Neutrophil

Neutrophils are pivotal immune cells within TME that exhibit both tumor-inhibiting and tumor-promoting actions, including the stimulation of tumor growth, angiogenesis, tissue invasion, and metastasis. Neutrophils can also undermine the immune system’s response to cancer by recruiting regulatory T cells and suppressing the activity of natural killer cells, highlighting their significance as potential therapeutic targets in the treatment of cancer (179, 180). Colorectal CSC-derived exosomes carry tri-phosphate RNAs which are capable of inducing the expression of IL-1β in neutrophils, sustaining their prolonged survival through a pattern recognition-NF-κB signaling axis (161). The primed neutrophils are subsequently attracted to the TME by CXCL1 and CXCL2, advancing the progression of colorectal cancer (161).

As the hallmarks of protumor N2 neutrophils, neutrophil extracellular traps (NETs) are networks composed of extracellular DNA fibers decorated with granule proteins that are released by neutrophils, which can trap and kill foreign pathogens (181–183). In recent years, it has been acknowledged that NETs play a tumor-promoting role in cancer and facilitate the progression and metastasis by trapping cancer cells (184, 185). The secreted factors or EVs of melanoma CSCs increase the formation of NETs, which in turn reinforce the stemness properties of CSCs (186). NETs are implicated in enhancing CSC-like features and fostering the transition to EMT state in breast cancer (162).

### MDSC

As immunosuppressive cells, MDSCs represent a heterogeneous population of immature cells recognized for their capacity to impede T cell responses and facilitate the advancement of cancer (187, 188).

MDSCs have the ability of increasing CSC population and promoting stemness properties. MDSCs could induce the formation of CSCs, sustain their survival and propagation, and enhance the metastatic growth of tumors (189). MDSCs could increase stemness of ovarian CSCs by inducing miRNA101 expression in CSCs (163). For breast cancer, elevated stem-like properties were observed with the IL-6-STAT3 pathway activation in MDSCs, and the degree of MDSC infiltration positively correlated with the number of CSCs (165). In pancreatic cancer, activation of pSTAT3 in MDSC could increase CSC population and promote EMT (164).

Current studies suggest that EVs originating from MDSCs and CSCs could serve as mutual catalysts, amplifying each other’s functional capabilities in a reciprocal manner. In colorectal cancer, MDSCs also sustain the survival and stemness of CSCs due to the exosomal S100A9 released from MDSCs (32). Glioma CSC-derived exosomes promote the presence of monocytic MDSCs, by stimulated the expression of arginase-1 and IL-10 in immature CD14+ monocytes (169).

### T cell

In immune defense against cancer, T cells, particularly CTLs, also cytotoxic CD8+ T cells, are critical for cancer detection and eradication. CD4+ T cells support this process by promoting the activation and growth of CD8+ T cells and can sometimes directly target tumor cells themselves, thus also playing a vital role in the efficacy of cancer immunotherapies (190).

CSC-derived EVs contribute to immune evasion in cancer by suppressing T cell functions. Renal CSC-derived EVs have been proven to significantly hinder T cell activation and proliferation, primarily owing to HLA-G secretion, contrast to EVs from non-stem renal cancer cells (160). The investigation into the impact of EVs derived from esophageal CSCs on T cell dynamics revealed that EVs overload O-linked β-*N*-acetylglucosamine transferase (O-GlcNAc transferase, OGT). Upon uptake by neighboring CD8+ T cells, OGT within these EVs leads to an increased expression of PD-1 in the T cells, and shield CSCs from immune-mediated destruction, thereby contributing to the immune evasion (166). In HNSCC, CSC-derived EVs specifically interact with PD1+ T cell, suggesting a direct involvement in modulating T cell behavior which plays a crucial role in cancer immune evasion mechanism (30). In addition, extracellular matrix protein tenascin-C in EVs from brain CSCs can inhibit T cell immunity (167). For colorectal CSCs, miRNA-146a-5p in their exosomes has a notable impact on the distribution of T cells in cancer patients. Specifically, patients with higher levels of serum exosomal miR-146a-5p showed fewer number of tumor-infiltrating CD8+ T cells (168)

CSC-derived exosomes have a selective impact on different T cell subtypes. In glioma, EVs from CSCs suppress activation, proliferation, and Th1 cytokine production in effector T cells, while regulatory T cells remain largely unaffected. Notably, these exosomes enhance the proliferation of CD4+ T cells, illustrating their complex role in modulating immune responses in glioma (169). However, the effects of exosomes released by CSCs on other subsets of T helper cells such as Th17 cells have yet to be understood.

## Developing EV-based anti-cancer immunotherapeutic strategies

EVs are favored for therapeutic delivery due to their superior biocompatibility and ability to penetrate biological barriers (191, 192). These years, EVs have been developed as targeted delivery systems to disrupt CSC functions by transporting RNA-based therapeutics into CSCs. EVs can be engineered to carry siRNAs that target key signaling pathways such as Wnt/β-catenin in liver cancer, leading to the suppression of CSC proliferation (193). In non-small cell lung cancer, EVs delivering APE1 shRNA have demonstrated potential in overcoming drug resistance (127). Furthermore, EVs designed to silence genes can reduce resistance to sorafenib treatments in liver cancer, promising to enhance patients’ clinical outcomes (194).

Notably, researchers have been making persistent efforts to develop engineered EVs as immunotherapeutic strategies to combat immunosuppressive state fueling by cancer cells and immune cells (159, 195–198). Xu et al. has developed a bispecific EVs (BsEVs) engineered from DCs that target tumor antigen CD19 on tumor cells and block the PD-1 checkpoint, thus bolstering cancer immunotherapy (197). These BsEVs show remarkable tumor-homing capabilities and can substantially remodel the tumor’s immune landscape, demonstrating their potential in personalized and versatile cancer treatments. Innovatively, chimeric exosomes, produced by M1 macrophage-tumor hybrid cells, naturally targeting to lymph nodes and tumors, enhancing T cell response, and overcoming immunosuppression. This dual-action immunostimulatory exosome strategy can alleviate tumors and enhance survival in animal studies and shows potential in personalized immunotherapy, especially when used with PD-1 inhibitors (199). Moreover, a dual-functional exosome delivery system that employs bone marrow mesenchymal stem cell-derived exosomes loading with galectin-9 siRNA and the immunogenic cell death trigger oxaliplatin, promises to enhance immunotherapy. This system aims to achieve win-win idea of both counteracting the immunosuppressive actions of M2 macrophages and simultaneously improving tumor targeting (200).

However, the immunotherapeutic approaches using EVs to target CSCs is still in its early stage and requires further in-depth and comprehensive research. Naseri et al. established a DC-based therapeutic strategy using colon CSC-derived exosomes as antigen sources, aiming to increase proliferation and activation of T cells specifically for killing CSCs (159). Besides leverage EVs for support, disrupting the interactions of CSC-derived EVs with macrophages emerges as a potential therapeutic choice. Colon CSC-derived exosomes, containing molecules like IL-6, p-STAT3, TGF-β1, and beta-catenin, are known to promote the generation of cancer-associated fibroblasts and M2 macrophages. Ovatodiolide, a bioactive compound, has been found to reduce these harmful components in exosomes, consequently weakening chemotherapy resistance (201). This suggest that ovatodiolide could serve as an effective agent against colon cancer through disrupting the exosomal supply CSCs provide to immunosuppressive cells.

Future directions for targeting CSCs and the tumor immune microenvironment are pointed to be focused on integrating EVs with cutting-edge cancer therapeutic strategies, such as differentiation therapy and synthetic lethality, aiming to provide more effective and precise cancer treatments. Differentiation therapy is an innovative approach that exploits the plasticity of CSCs by inducing them to differentiate into less malignant, more differentiated cells, making them more susceptible to cytotoxic drugs (202, 203). Acting as biocompatible natural carriers, EVs are promising to be engineered to deliver differentiation-inducing and immune activating molecules to target CSCs and immune microenvironment, thereby synergistically eliminating refractory CSC pool. Synthetic lethality (SL) is defined as the simultaneous inactivation of two genes lead to cell death, whereas the loss of either gene alone is not lethal (204, 205). A prime example is the use of PARP inhibitors in combination with BRCA1/2 mutations, which results in the targeted death of cancer cells (206). Multifunctional engineered EVs present a promising delivery mechanism for SL-based therapy, as co-delivering multiple therapeutic agents that target separate pathways essential CSC survival and immune responding within the same EVs can enhance the efficacy of SL approaches. To conclude, EV-based therapies offer a versatile and targeted approach to treating cancer by addressing both CSCs and the tumor immune microenvironment.

## Discussion

CSCs have been recognized as crucial targets in oncology due to their irreplaceable roles in tumor growth, metastasis, and the potential for developing more effective cancer therapies by disrupting CSC-specific pathways. The interaction between CSCs and immune cells significantly influences cancer progression, offering therapeutic avenues to modify the immune environment and exploit immune cells for CSC eradication. EVs, as masters of intercellular communication, not only shed light on the complex dynamics of cell interactions but also offer platforms for bioengineering as cutting-edge immunotherapeutic tools, harnessing their natural communication ability to modulate immune responses and precisely combat against CSCs. Specifically, compared with EVs derived from non-malignant stem cell such as mesenchymal stem cells which are known for regenerative and anti-inflammatory properties (207, 208), CSC-derived EVs possess superior cancer therapeutic capacities due to their inherent tumor-targeting specificity, tumor immune modulation activity, and higher uptake efficiency by cancer cells (209). These characteristics makes CSC-derived EVs exceptionally advantageous as potential mediators for cancer therapeutic payload delivery.

In this review, we have provided current research on EV-mediated communications of CSCs with individual subtype of immune cells in a wide spectrum of cancers. In terms of clinical translation, it is noteworthy that preclinical investigations into ovatodiolide have highlighted its potential as a disruptor of the deleterious feedback loop between CSCs and immunosuppressive macrophages, heralding an augmentation in the therapeutic efficacy for patients with colon cancer undergoing chemotherapy.

Despite the advancements discussed in our review, it is critical to emphasize that our understanding of the interactions between CSCs, EVs, and immune cells is still in its infancy. Especially understudied are the roles of NK cells, Th17 cells, and B cells in this tripartite communication, which is surprising given their crucial roles in the immune response to cancer. Take NK cells for example, as cytotoxic lymphocytes in the innate immune system, they possess the ability to eliminate cells infected by viruses or cancer cells (210). NK cells are powerful in cancer immunotherapy because of being able to swiftly attack cancer cells, thereby boosting both the immediate and long-term immune defense against tumors (211, 212). Previous studies (213–216) have found that while CSCs with reduced MHC-I expression and certain CSC markers can activate NK cells’ cytotoxic functions, leading to their effective elimination in various cancers, CSCs also employ numerous mechanisms to suppress NK cell-mediated immune responses, such as downregulating activation ligands or entering a dormant state to evade detection. This intricate dynamic between NK cells and CSCs suggests a potential role for EVs in mediating their interactions. Recognizing this, we advocate for a concerted effort to deepen the investigation into CSC-EV-immune cell interactions. Furthermore, the field of CSC research necessitates to advance precise isolation techniques that consider the heterogeneity of CSCs, including the development of comprehensive identification strategies including refined cell surface markers. Timely initiation of preclinical and clinical trials, grounded in laboratory findings, is imperative to substantiate the therapeutic efficacy and expedite the translation of research into improving patient survival and quality of life.

## Author contributions

YJ: Writing – original draft, Writing – review & editing. CZ: Writing – review & editing. WY: Conceptualization, Supervision, Writing – review & editing. XL: Conceptualization, Funding acquisition, Project administration, Supervision, Writing – review & editing.
